# Supplementary material for: Single-nucleotide conservation state annotation of the SARS-CoV-2 genome
Source: Commun Biol. 2021 Jun 3;4:698. doi: 10.1038/s42003-021-02231-w (PMC8175581; doi:10.1038/s42003-021-02231-w)
Supplement: Supplementary file 2 — Supplementary Information [file 42003_2021_2231_MOESM2_ESM.pdf]

Supplementary Tables

1. Summary of grouping, align and match probabilities, and notable enrichments of ConsHMM conservation states learned from the Sarbecovirus alignment.
2. Summary of grouping, align and match probabilities, and notable enrichments of ConsHMM conservation states learned from the vertebrate CoV alignment.
3. Genomic segments unique to pathogenic human CoV and missing in less pathogenic human CoV identified by state V14.

Supplementary Figures

1. Conservation state enrichment for protein products.
2. Sarbecoviruses associated with states S12 and S13 in the phylogenetic tree of the 44-way Sarbecovirus alignment.
3. Precision-recall plots for predicting genes and regions of interest.
4. Conservation states' relationship to PhastCons and PhyloP annotations.
5. Vertebrate CoV associated with states V10 and V11 in the phylogenetic tree of the vertebrate CoV alignment.
6. Conservation state enrichment for SARS-CoV-2 mutations.
7. Correlation with measured mutational effect for tracks based on state depletion of mutations and existing sequence constraint scores.

Description	*State*	*Aligns to*	*Matches to*	*Notable enrichments*
Unique to SARS-CoV-2 and RaTG13	S28	RaTG13	RaTG13	Most enriched for human ACE2 binding motif; Enriched for nonsingleton mutations
Aligns to most and matches to Sarbecoviruses closely related to SARS-CoV-2	S9	All Sarbecoviruses	RaTG13	
	S6			Enriched for nonsingleton mutations and homoplastic mutations
	S7		Small subset of close strains including RaTG13	
	S8			Most enriched for heptad repeat 1
	S10		Subset of strains including RaTG13 and SARS-CoV	Most enriched for spike protein's receptor binding domain (RBD) and gene ORF3a
Deviation along a branch of the Sarbecovirus phylogeny	S12	All Sarbecoviruses	Subset of strains corresponding to a subtree in the phylogeny (**Supplementary Fig. 2**)	Enriched for nonsingleton mutations
	S13			
Aligns to most and matches to a subset of Sarbecoviruses	S16	All Sarbecoviruses	Distinct subsets of strains with varying distance to SARS-CoV-2	
	S11			
	S15			
	S5			Most enriched fusion peptide 1
	S24	All except several distal strains		Most enriched for gene ORF8
Aligns and matches to most Sarbecoviruses	S4	All Sarbecoviruses	All except several strains	
	S3			
	S2			
	S1			
	S26			Enriched for nonsingleton mutations
	S21			
	S22		All except a strain	
	S23			Most enriched for gene S
	S14		All Sarbecoviruses	
	S17			Depleted of nonsingleton mutations and homoplastic mutations
	S18			Most enriched for gene E and region that interacts with RMP Remdesivir; Most depleted of nonsingleton mutations
	S20			
	S19		All except two distal strains	Most enriched for genes ORF6, ORF7a, and N and RNA-binding region; Enriched for nonsingleton mutations
	S25	All except two distal strains	All except two distal strains	Most enriched for gene ORF7b
	S27	All except two strains	All except two strains	Most enriched for genes orf1a (YP_009725295.1) and orf1ab (YP_009724389.1)
Non-coding or putative artifact	S29	All except several close and distal strains	All except several close and distal strains	Most enriched for intergenic bases, gene ORF10; Most enriched for nonsingleton mutations
	S30			

Supplementary Table 1. Summary of grouping, align and match probabilities, and notable enrichments of ConsHMM conservation states learned from the Sarbecovirus alignment.

First column contains each group's description, where a group consists of one or more states based on the hierarchical clustering of emission parameters as explained in **Fig. 2a**. Second column contains the state identifiers. Third and fourth columns describe the strains for which each state has align and match probabilities greater than 0.5, respectively. The last column summarizes notable enrichment of external annotations, as shown in **Fig. 2b**. RaTG13 refers to a bat CoV most closely related to SARS-CoV-2. Nonsingleton mutations mentioned in this table are nonsingleton mutations observed in SARS-CoV-2 sequences based on Nextstrain's annotation of GISAID's SARS-CoV-2 sequences[1,2] (**Methods**). Homoplastic mutations mentioned in this table are stringently identified homoplastic mutations from a previous study[3]. All enrichment and depletion reported here have a two-sided binomial test p-value significant at a 0.05 after Bonferroni correction.

Description	*State*	*Aligns to*	*Matches to*	*Notable enrichments*
Aligns and matches to four closest strains –two bat CoV (RaTG13 and BM48-31/BGR/2008), pangolin CoV, and SARS-CoV	V22	Four closest strains and several others	Four closest strains and several others	
	V28	Four closest strains except pangolin CoV	Four closest strains except pangolin CoV	Most enriched for gene ORF3a
	V29	RaTG13 and SARS-CoV	RaTG13	
	V30	RaTG13 and pangolin CoV	RaTG13 and pangolin CoV	Most enriched for nonsingleton mutations; Most enriched for genes ORF7b and ORF8
	V20	Four closest strains	RaTG13 and pangolin CoV	Enriched for nonsingleton mutations; Most enriched for receptor binding domain (RBD) and human ACE2 binding domain motif
	V19		Four closest strains	Most enriched for genes ORF6 and ORF7a
	V18	Four closest strains and several others	Four closest strains	
	V16			
	V17		Four closest strain and a bat CoV	
	V21			
	V15		Four closest strains	Most enriched for intergenic bases and genes E and ORF10
Aligns and matches to about half of the strains, particularly to four closest strains	V14	Up to half of strains, most close to SARS-CoV-2	Up to half of strains, most close to SARS-CoV-2	Most enriched for dimerization-associated region
	V13			Most enriched for gene N and RNA-binding region; Enriched for nonsingleton mutations and homoplastic mutations
	V23			
	V24			Most enriched for gene M
	V12			Most enriched for gene S and heptad repeat 2
	V25			Most enriched for gene orf1a (YP_009725295.1)
Aligns to most and matches to some vertebrate CoV	V9	All except several strains	Four closest strains	
	V8			Most enriched for orf1ab (YP_009724389.1)
	V3	All vertebrate CoV		Most enriched for fusion peptide 1; Enriched for nonsingleton mutations
	V2			Most enriched for heptad repeat 1
	V6		Four closest strains and several distal strains, most of which are from birds	
	V5		Four closest strains with several others	
	V4			Most enriched for fusion peptide 2
Aligns to all and matches to most vertebrate CoV	V1	All vertebrate CoV	All except several close strains	
	V7		All vertebrate CoV	
	V27			Depleted of nonsingleton mutations
Aligns and matches to most except some CoV with avian hosts	V26	All vertebrate CoV	All except several CoV, most of which are from birds (**Supplementary Fig. 5**)	
	V11	All except several CoV, most of which are from birds (**Supplementary Fig. 5**)		Most depleted of nonsingleton mutations
	V10			Depleted of nonsingleton mutations

Supplementary Table 2. Summary of grouping, align and match probabilities, and notable enrichments of ConsHMM conservation states learned from the vertebrate CoV alignment.

Similar to **Supplementary Table 1** except showing vertebrate CoV model's states instead of Sarbecovirus model's states.

start	end	gene	confirmed based on human CoV	Gussow et al.
7390	7450	orf1ab		
7807	7809	orf1ab		
7809	7816	orf1ab	TRUE	
7816	7825	orf1ab		
7868	7871	orf1ab		
7931	7933	orf1ab		
8575	8589	orf1ab		
8640	8647	orf1ab		
8658	8660	orf1ab		
8888	8892	orf1ab		
8892	8893	orf1ab	TRUE	
8893	8899	orf1ab		
8963	8968	orf1ab		
8969	8973	orf1ab		
10237	10238	orf1ab		
10797	10799	orf1ab		
10869	10871	orf1ab		
11074	11076	orf1ab		
11370	11371	orf1ab		
12912	12913	orf1ab		
13328	13331	orf1ab	TRUE	
16190	16193	orf1ab		
18171	18174	orf1ab		
18230	18231	orf1ab		
19131	19134	orf1ab		
19958	19961	orf1ab		
20351	20353	orf1ab		
20391	20397	orf1ab		
23843	23844	S		
23938	23941	S		
24001	24002	S		
24226	24227	S		
24227	24229	S	TRUE	TRUE
24775	24778	S		
24990	25000	S		
25322	25345	S		
26610	26611	M		
26874	26938	M		
26939	27041	M		
27043	27047	M		
27049	27067	M		
27078	27085	M		
27086	27135	M		
28396	28415	N		
28415	28423	N	TRUE	
28496	28500	N		
28561	28567	N		
28680	28686	N		
28704	28706	N		
28797	28809	N		
28857	28875	N		
28946	28966	N		
29001	29002	N		
29012	29014	N		
29024	29026	N		
29115	29116	N		TRUE
29116	29124	N	TRUE	TRUE
29218	29233	N		
29241	29362	N		
29374	29400	N		
29730	29731	non-coding		
29764	29771	non-coding		
29784	29803	non-coding		

Supplementary Table 3. Genomic segments unique to pathogenic human CoV and missing in less pathogenic human CoV identified by state V14.

Each row corresponds to a genomic segment annotated by state V14, which corresponds to bases with high (>0.5) align probabilities for SARS-CoV and MERS-CoV and low (<0.5) align probabilities for common-cold-associated human CoV. First and second columns denote 0-based genomic coordinates (BED format). Third column shows the gene in which the genomic segments are located if it is in a gene or “non-coding” if it is not a gene. Fourth column denotes whether the base is confirmed to be in all pathogenic human CoV and missing in all less pathogenic human CoV based on an alignment of 944 human CoV sequences. Last column denotes whether the genomic segment was identified as an insertion specific to pathogenic strains in a prior study[4].

a Enrichment for protein products in states learned from the Sarbecovirus alignment

State	nsp1	pp1a	pp1ab	nsp2	nsp3	nsp4	3CL-PRO	nsp6	nsp7	nsp8	nsp9	nsp10	Pol	nsp11	Hel	ExoN	nsp15	nsp16	S glycoprotein	Spike protein S1	Spike protein S2	Spike protein S2'	ORF3a	E protein	M protein	ns6	ORF7a	ns7b	ns8	NC	ORF9b	ORF14
S1	0.6	1.2	1.1	1	1.4	1	0.9	0.8	0.2	0.3	0.9	0.8	1.3	0	1	0.9	1	1	0.6	0.4	0.9	1.1	1.9	0.2	0.6	0.9	0.8	1.7	0.3	0.5	0.6	0.7
S2	0.9	0.9	1.3	0.3	0.9	1	1.4	0.4	0	0.5	0.5	0.8	2.1	0	2.3	3.1	1.5	0.4	0.1	0.1	0.2	0.2	0.2	0	0.2	0	0.5	0	0.5	0.4	1.6	0
S3	0.9	1.8	1.3	2.2	2.4	1.1	1.9	1.3	0.1	0.1	0.4	0	0.1	0	0.1	0.2	1.1	0.8	0.3	0.2	0.4	0.3	0.3	0	0.4	0.7	0.8	0	0.8	0.6	0.9	1.2
S4	1.5	1	1	1.2	1.1	0.9	0.7	0.5	0.3	0.1	0.5	1.4	0.7	0	0.9	0.8	1.4	1.3	1.4	1.3	1.5	1.6	0.8	0.4	0.5	0.2	0.7	0	2.2	1.2	1.4	1.7
S5	0.2	0.5	0.6	0.7	0.6	0.4	0.4	0.3	0	0.1	1.1	0.2	0.5	0	0.7	0.5	0.9	2.1	3.9	5.2	2.4	2	0.8	0	1.7	0	0.3	0	0	0.1	0	0
S6	0.5	0.8	1	0.6	0.8	0.6	0.5	0.7	0.5	0.4	1.7	1.3	1.4	2.9	1.1	1.9	1.9	0.6	1.3	1.6	1	0.9	1	0	0.5	0.5	0.7	0	0.3	0.9	0.6	0.3
S7	1.6	1.7	1.1	2.7	2	2	1.1	1.5	0	0	0	0	0	0	0	0	0.2	0.8	0.4	0.3	0.5	0.5	1.5	0.5	1.3	2.2	1	1.7	0.7	0.6	0.5	1.3
S8	0.7	1.2	1.1	1.5	1.3	1.3	1.1	1	0.4	0.1	1.4	0.3	0.6	0	1	1	0.8	0.8	1	0.8	1.2	1.3	2.1	0.3	0.9	0.3	0.9	1.4	0.4	0.5	0.4	0
S9	0	0.2	0.1	0	0.4	0	0	0	0	0	0	0	0	0	0	0	0	0	3.8	7.2	0	0	0	0	0	0.9	0	0	27	0	0	0
S10	0	0	0	0.1	0	0	0	0	0	0	0	0	0	0	0.1	0	0	0.5	6.6	9.3	3.4	2.3	4.5	0.7	0.2	0.8	0	0	0	0	0	0
S11	0.4	0.5	0.7	1	0.5	0.4	0.1	0.3	0.2	0	0.6	0.5	0.4	0	1.1	1.3	1.4	1.3	3.4	4.4	2.4	2.2	0.8	0.5	0.5	1.8	0.3	0	0	0.4	0.4	0
S12	1	1.3	1.1	1.7	1.3	1.4	1	1.1	0.5	0.2	0.4	0.7	0.8	0	0.5	1.4	1	0.7	1.1	1.1	1.2	1	0.3	0.3	0.2	1.1	0.9	0	0.2	0.6	0.9	0.3
S13	0.4	1.6	1.3	1.4	1.8	1.8	1.3	1.7	0.2	0.3	1.2	1.3	0.7	0	1.1	0.8	1	0.7	0.4	0.2	0.5	0.5	0.3	0	0.1	0	0	0	0.2	0.3	0.5	0.6
S14	1	0.7	0.9	1.2	0.7	0.3	0.4	0.8	0.3	0.1	0.3	0.5	0.9	0	1	1.2	2	2.5	1.5	1.8	1.2	1.1	1.2	1	1.2	1.2	2.1	0	0	0.9	0.4	2
S15	0	0	0	0	0.1	0	0	0	0	0	0	0	0	0	0	0	0	0	4.4	8.2	0	0	0	0	0	0	0	0	33	0	0	0
S16	0	0.1	0.1	0	0.1	0	0	0	0	0	0	0.4	0	0	0	0	0	1.6	6.5	7.3	5.5	3.8	3.8	0	0	0	0	0	0	0	0	0
S17	1	1	1.1	0.9	0.9	1.2	1.2	1.2	0.3	0.3	1	1.1	1	0.4	1.3	1.2	1.2	1.3	0.9	0.7	1.2	1.2	0.9	0.6	1.2	0.9	0.9	0.8	0	1.1	1	0.9
S18	1.2	0.6	1	0	0.1	0.4	1.1	0.9	0.3	0.8	3.5	3.1	3.4	7.6	1.1	1.1	0.7	0.3	0.1	0	0.2	0.3	0.9	7.5	1.3	0.4	0.2	0.9	0	1.7	2.2	1.9
S19	1.7	1.1	0.9	1.5	1.1	0.9	0.6	0.9	0.1	0.1	0.5	0.4	0.7	0.8	1	0.7	0.9	0.6	0.7	0.5	1	0.9	1.3	0.9	1.4	3.9	3.8	3.1	0	1.9	2	2.6
S20	1.2	1	1.2	1	1	0.9	0.8	1	0.5	0.3	0.3	1.4	1.5	0	1.3	1.8	1	1.2	0.7	0.5	0.8	1	0.8	0.5	0.6	0.3	1	0.4	0	0.7	0.7	0.2
S21	2.3	1.1	1	1.4	0.9	2	0.3	0.3	0	0.3	0	1.8	0.3	6.6	1.4	0.6	0.7	1.4	1.7	2.4	1	1.3	0.6	0	0	0	2.4	2	0	1	0.9	0
S22	1.9	1.3	1.1	2.2	1.1	1.3	1.5	0.5	0.4	0.4	0	1.3	0.5	0	1.2	0.9	1.2	0.8	0.7	0.5	1.1	1.1	0.5	0	0.6	0.6	1.6	0.8	0	0.8	1.1	1.4
S23	0	0	0	0	0.1	0	0	0	0	0	0	0	0	0	0	0	0	0	7.3	13	0.4	0.3	0.5	0	0	0	0	0	0	0	0	0
S24	0	0.2	0.1	0	0.5	0	0	0	0	0	0	0	0	0	0	0	0	0	2.3	4.3	0	0	0	0	0	0	0	0	47	0.3	0	0
S25	0	0.8	0.5	0.7	1.5	0	0	0	0	0	0	0	0	0	0	0	0	0	0.2	0.3	0.2	0.3	1.8	0	0	1.5	3	16	26	1.1	1.4	1.2
S26	0.8	1	0.8	1	1.2	1.6	1.6	0.8	0.1	0	0.7	0	0.2	0	0.6	0.2	0.8	1.7	1.3	1	1.6	1.6	2.1	0.7	1.8	1.4	3.2	2	0	1.7	2.9	1.5
S27	0	2.2	1.3	0	4.9	0	0	0	0	0	0	0	0	0	0	0	0	0	0	0	0	0	0	0	0	0	0	0	0	0	0	0
S28	0	0.8	0.5	0.6	1.6	0	0	0	0	0	0	0	0	0	0	0	0	0	3.7	7	0	0	0.4	0	0.5	0	0	0	1.6	0	0	0
S29	0	0	0	0	0	0	0	0	0	0	0	0	0	0	0	0	0	0	0	0.1	0	0	0	0	0	0	0	0	0	0	0	0
S30	0	0	0	0	0	0	0	0	0	0	0	0	0	0	0	0	0	0	0	0	0	0	0	0	0	0	0	0	0	0	0	0
Coverage	1.8	44	71	6.4	19	5	3.1	2.9	3.3	7.9	1.1	1.4	9.3	0.1	6	5.3	3.5	3	13	6.7	5.9	4.6	2.8	0.8	2.2	0.6	1.1	0.4	1.1	4.2	1	0.7

b Enrichment for protein products in states learned from the vertebrate CoV alignment

State	nsp1	pp1a	pp1ab	nsp2	nsp3	nsp4	3CL-PRO	nsp6	nsp7	nsp8	nsp9	nsp10	Pol	nsp11	Hel	ExoN	nsp15	nsp16	S glycoprotein	Spike protein S1	Spike protein S2	Spike protein S2'	ORF3a	E protein	M protein	ns6	ORF7a	ns7b	ns8	NC	ORF9b	ORF14
V1	0	0.3	1.3	0	0	0	1.9	0.6	0	0.2	0.4	3.6	2.9	0	3.1	3.1	2.6	2.4	0.6	0	1.2	1.5	0	0	0	0	0	0	0	0	0	0
V2	0	0.3	1.2	0	0	0	1.7	0.6	0.1	0.1	0.7	3.2	2.7	0	2.8	2.3	2.7	1.9	1.3	0	2.8	3.6	0	0	0	0	0	0	0	0	0	0
V3	0	0.4	1.3	0	0	0	2	1.2	0	0.1	0.9	3.6	3.3	1.6	2.9	2.8	2.3	2.1	0.5	0	1.1	1.4	0	0	0	0	0	0	0	0	0	0
V4	0	0.4	1.3	0	0	0	1.7	1.3	0	0.2	0.9	3.3	2.8	1.7	3	2.8	2.6	2	0.8	0	1.8	2.3	0	0	0	0	0	0	0	0	0	0
V5	0	0.5	1.3	0	0	0.8	2	0.9	0.5	0.5	0.5	1.9	3	1.6	3	2.1	2.6	2.2	0.4	0	0.9	1.2	0	0	0	0	0	0	0	0	0	0
V6	0	0.5	1.4	0	0	0	3.4	0.4	0.1	0.3	1.2	3.2	2.6	0	3.2	2.8	4.2	1.7	0.1	0	0.3	0.4	0	0	0	0	0	0	0	0	0	0
V7	0	0.3	1.3	0	0	0	2	0.7	0.1	0.3	0.8	2.8	3	0.7	2.8	2.6	2.8	2.5	0.7	0	1.5	2	0	0	0	0	0	0	0	0	0	0
V8	0	1.6	1.4	0	0.3	1.4	2.9	3.7	2.7	2.6	7.2	0.2	0.6	0	0.3	1.9	0.1	3.7	0	0	0	0	0	0	0	0	0	0	0	0	0	0
V9	0	1.9	1.4	0	0.7	0.1	4.7	15	0.7	0.3	4.9	0.3	0.3	11	0.1	0.7	1.5	1.6	0	0	0.1	0.1	0	0	0	0	0	0	0	0	0	0
V10	0	1.5	1.4	0	0.4	0.9	3.8	4.2	1.9	2	8	0.1	0.5	1.7	0.6	1.5	1.4	3.2	0.1	0	0.1	0.1	0	0	0	0	0	0	0	0	0	0
V11	0	1.5	1.3	0	1	1.6	3.1	1.6	1.8	1.5	4.9	0.6	0.4	2.6	0.2	1.3	0.7	3.6	0.3	0	0.6	0.7	0	0	0	0	0	0	0	1.1	0	0
V12	0	0.8	0.6	0	1.6	0.1	0.1	1.2	0.3	0.1	0.9	0	0.1	0	0.1	0.1	1.3	0	3.8	0	8.2	10	0	0	0	0	0	0	0	1.8	0.2	0
V13	0	0.8	0.5	0	0.8	2.8	0.4	0.6	0	0	0.1	0	0.2	4.9	0.1	0.3	0.3	0.3	0.2	0	0.5	0.2	0	0	0	0	0	0	0	14	10	26
V14	0	0.4	0.3	0	0.5	1.1	0.2	0.1	0	0	0.1	0.3	0	0	0	0.2	0.4	0	0.4	0	0.9	1.1	0	0	14	0	0	0	0	8.2	4.8	6.2
V15	0	1.3	0.8	1.3	2.5	0	0	0	0	0	0	0	0	0	0	0	0	0	0.9	1.4	0.3	0.3	0	11	1.6	0	0	0	0	1.4	1	0
V16	0	1.3	0.8	0	2.7	0.5	0.1	0.5	0	0	0	0	0	0	0.1	0	0.4	0	2.2	2.8	1.4	1.5	0	0	1.2	0	0	0	0	0.2	0.7	0
V17	0	1.9	1.2	0	4.4	0	0	0	0	0	0	0	0	0	0	0	0	0	0.8	1.5	0	0	0	0	0.5	0	0	0	0	0.6	0	0
V18	0	1.7	1.1	2.1	3.3	0	0	0	0	0	0	0	0	0	0	0	0	0	1.8	3.4	0	0	0	0	0	0	0	0	0	0	0	0
V19	8.7	1.1	0.7	3.6	0.4	0	0	0	0	0	0	0	0	0.5	0	0	0	0	1.9	3.5	0	0	2.3	0.2	0	7.9	8.3	7.4	0	0.6	1.5	0
V20	4.8	1.1	0.7	4.4	0.6	0	0	0	0	0	0	0	0	0	0	0	0	0	2.8	5.2	0	0	2.1	0	0	5.4	5.1	3	0	0.2	0.6	0
V21	0	1.3	0.8	1.6	2.4	0	0	0	0	0	0	0	0	0	0	0	0	0	1.4	2.2	0.5	0	5.8	1.5	0.1	0	0	0	0	0.4	1.7	0
V22	0	2.1	1.3	0	4.7	0	0	0	0	0	0.3	0	0	0	0	0	0	0	0.1	0	0.3	0.1	0	0	1.9	0	0	0	0	0.5	0	0.7
V23	0	0.8	0.8	0	0.1	0.4	1.7	6.3	1	0.3	2.8	1	0.3	0	0.6	1.1	2.5	0.1	2.6	0	5.5	3.1	0	0	0	0	0	0	0	0.1	0.3	0
V24	0	0	0	0	0	0	0	0	0	0	0	0	0	0	0	0	0	0	0	0	0	0	0	0	40	0	0	0	0	2.8	12	0
V25	0	2.1	1.3	0	1.7	12	0	0	0	0	0	0	0	0	0	0	0	0	0.5	0	1.1	0	0	0	0	0	0	0	0	0	0	0
V26	0	0.4	1.4	0	0	0	1.8	0.7	0.2	0.3	1	3	3.1	1.1	3.2	3.1	2.8	3.3	0	0	0	0	0	0	0	0	0	0	0	0	0	0
V27	0	0.3	1.3	0	0	0	1.6	0.6	0.1	0.2	0.8	2.9	3.4	3.5	3.4	2.6	2	2.2	0.5	0	1.1	1.4	0	0	0	0	0	0	0	0	0	0
V28	0	1.6	1	8.2	1	0	0	0	0	0	0	0	0	0	0	0	0	0	0.7	1.2	0.1	0.1	7.2	0	0	0	0	0	0	0.2	0	0
V29	0	1.7	1.1	2.8	3	0	0	0	0	0	0	0	0	0	0	0	0	0	0.4	0.8	0	0	0.2	0	0	0	0	0	0	0	0	0
V30	0	0.2	0.2	0.1	0.5	0	0	0	0	0	0	0	0	0	0	0	0	0	1.8	3.4	0	0	0	0	0	2.7	1.3	8.3	45	0.1	0.5	0
Coverage	1.8	44	71	6.4	19	5	3.1	2.9	3.3	7.9	1.1	1.4	9.3	0.1	6	5.3	3.5	3	13	6.7	5.9	4.6	2.8	0.8	2.2	0.6	1.1	0.4	1.1	4.2	1	0.7

Supplementary Figure 1. Conservation state enrichment for protein products.

a. Fold enrichment for protein products in conservation states learned from the Sarbecovirus model. Each row corresponds to a state. First column contains the state ID. The following columns contain fold enrichment values for different protein products listed at the top of each column. Protein product coordinates and names were from UniProt Protein Product annotation[5]. Last row reports genome coverage percentage of each protein. Each cell corresponding to an enrichment value is colored based on its value with blue as 0 (annotation not overlapping the state), white as 1 to denote no enrichment (fold enrichment of 1), and red as the maximum enrichment value in this table. Each cell corresponding to a coverage percentage is colored based on its value with white as minimum and green as maximum.

b. Similar to **a**, except based on states learned from the vertebrate CoV model.

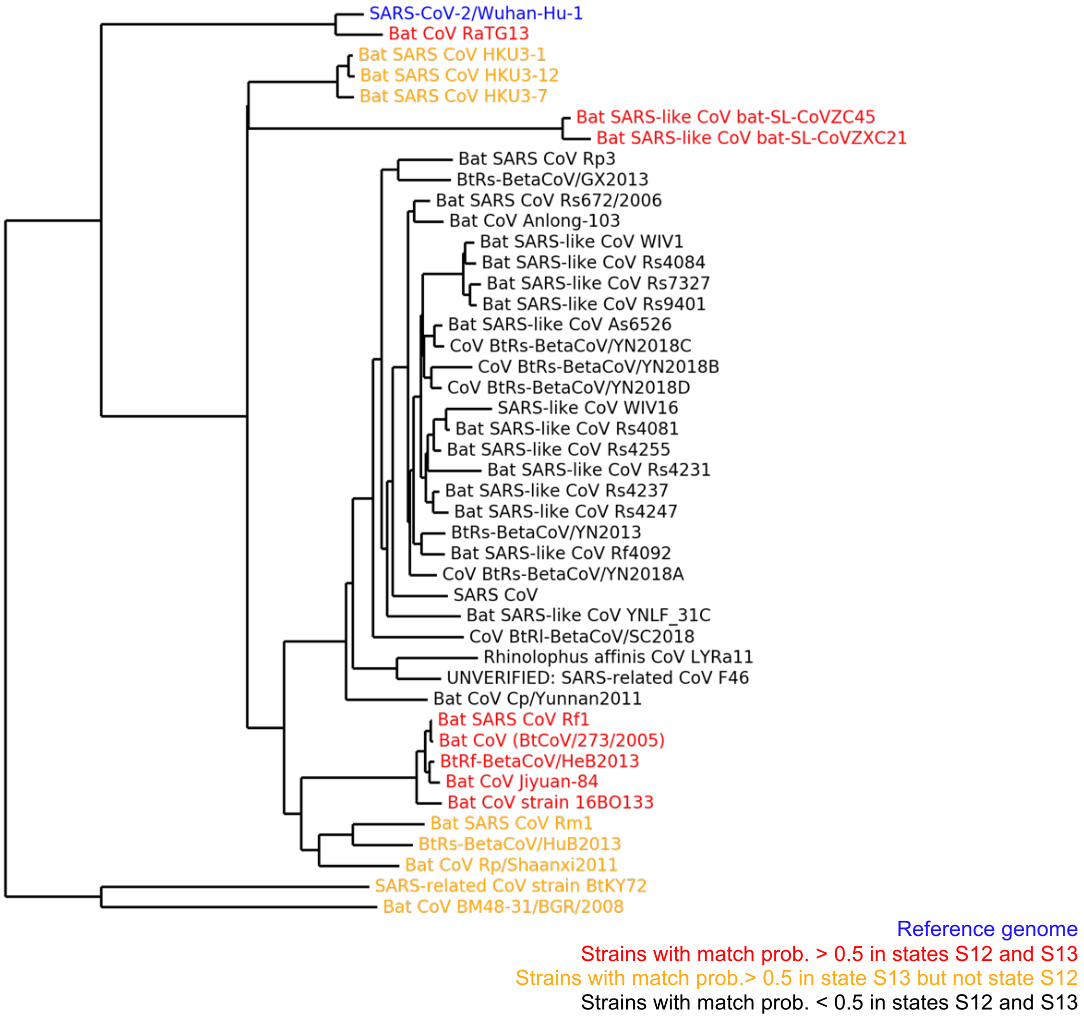

Supplementary Figure 2. Sarbecoviruses associated with states S12 and S13 in the phylogenetic tree of the 44-way Sarbecovirus alignment. Similar to **Fig. 2c** except strains colored according to their align and match probabilities in states S12 and S13. The strain colored in blue is the reference SARS-CoV-2 strain of the alignment, SARS-CoV-2/Wuhan-Hu-1. Strains colored in black are those that have match probabilities below 0.5 for both states S12 and S13. Strains colored in red are those with match probabilities above 0.5 for both states S12 and S13. Strains colored in yellow are those with match probabilities above 0.5 for state S13 but not for state S12. All strains have high (>0.95) align probabilities for states S12 and S13. States S12 and S13 are likely to correspond to a deviation along the branch preceding all strains colored in black.

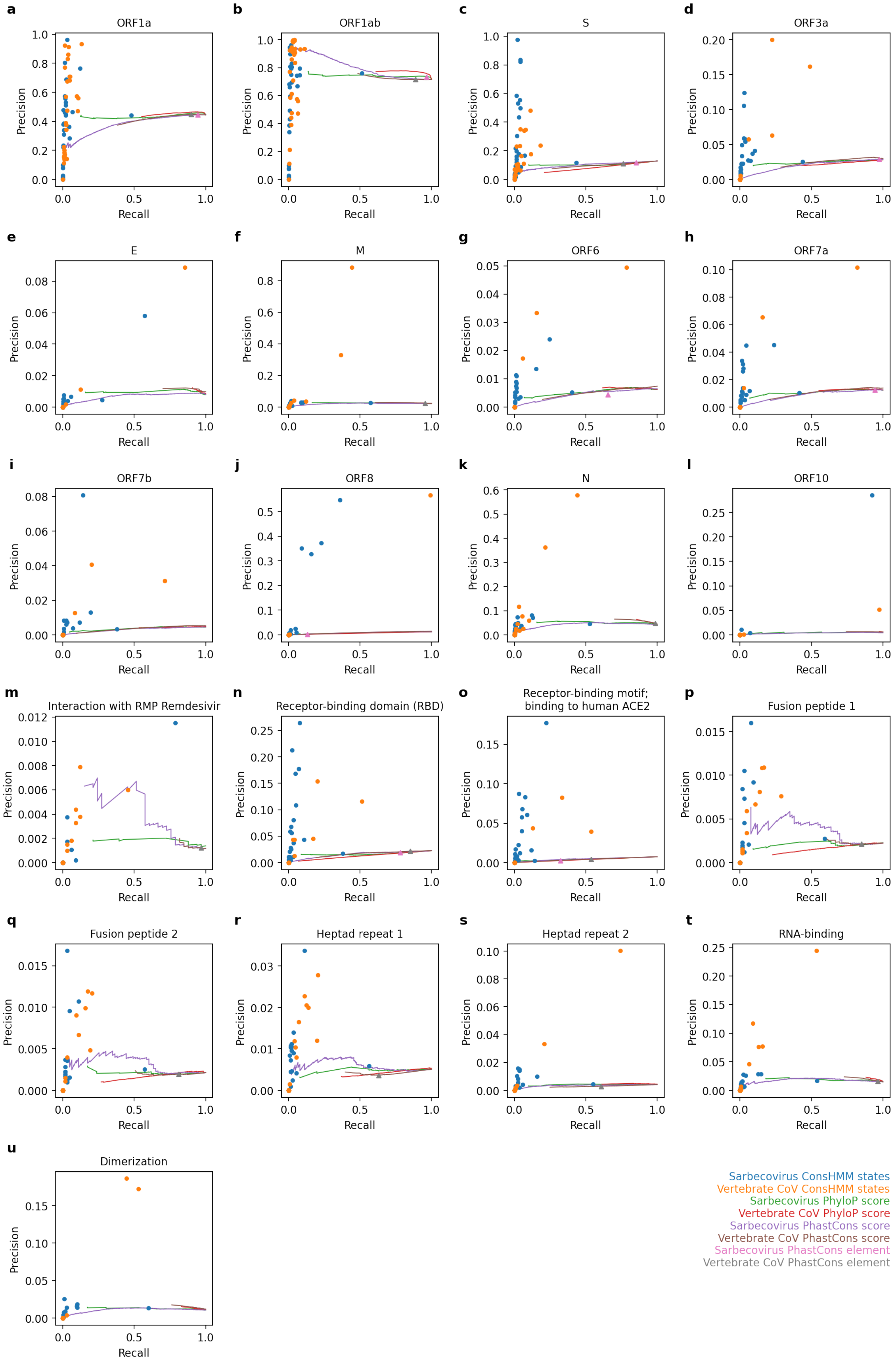
a
ORF1a
b
ORF1ab
c
S
d
ORF3a
e
E
f
M
g
ORF6
h
ORF7a
i
ORF7b
j
ORF8
k
N
l
ORF10
m
Interaction with RMP Remdesivir
n
Receptor-binding domain (RBD)
o
Receptor-binding motif; binding to human ACE2
p
Fusion peptide 1
q
Fusion peptide 2
r
Heptad repeat 1
s
Heptad repeat 2
t
RNA-binding
u
Dimerization
Precision
Recall
Sarbecovirus ConsHMM states
Vertebrate CoV ConsHMM states
Sarbecovirus PhyloP score
Vertebrate CoV PhyloP score
Sarbecovirus PhastCons score
Vertebrate CoV PhastCons score
Sarbecovirus PhastCons element
Vertebrate CoV PhastCons element

Supplementary Figure 3. Precision-recall plots for predicting genes and regions of interest. Shown in each subplot is a precision-recall plot for predicting bases that overlap external genomic annotations using ConsHMM conservation states and sequence constraint annotations. Above each subplot is the target annotation, which is either a gene (**a-l**) or a region of interest defined by UniProt[5] (**m-u**). In each subplot, prediction based on ConsHMM conservation states for bases overlapping the target annotation is shown with circles. Prediction based on sequence constraint scores is shown with continuous lines. Prediction based on PhastCons element is shown with triangles. Circles, lines, and triangles are colored according to the bottom right legend. Y-axis varies from subplot to subplot because the target annotations have different genome coverage. In most cases, at least one of the ConsHMM states have substantially greater precision at the same recall level than other sequence constraint annotations, suggesting that it has greater correspondence with the annotated bases.

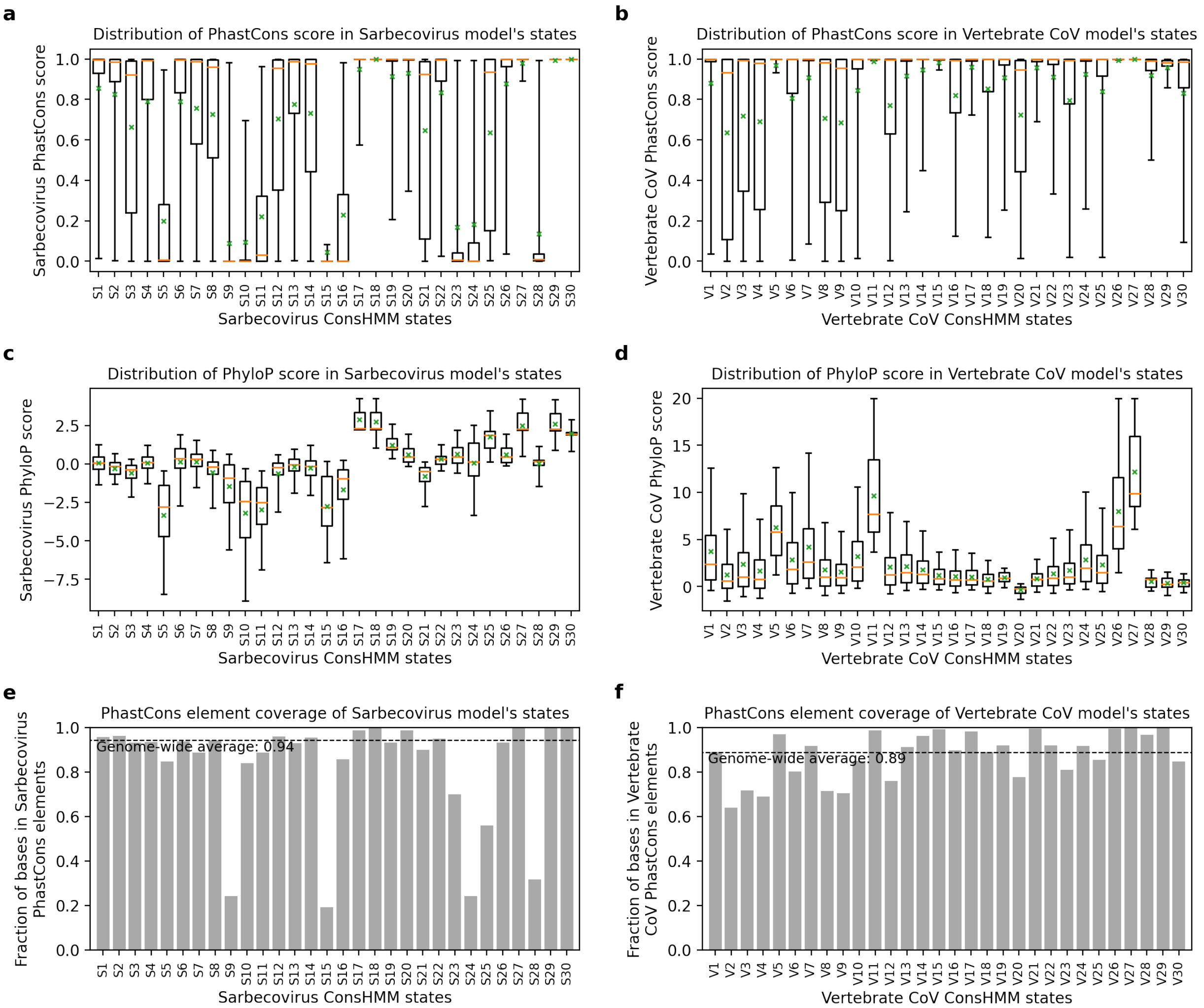

Supplementary Figure 4. Conservation states' relationship to PhastCons and PhyloP annotations.

a. Shown for each conservation state learned from the Sarbecovirus alignment (x-axis) is the distribution of PhastCons score learned from the same alignment (y-axis) in bases overlapping the state. Each distribution is represented by a boxplot with median (orange horizontal line), mean (green 'x'), Q1 and Q3 (box), and Q1–1.5 IQR and Q3+1.5 IQR (whisker), where Q1 and Q3 represent 25th and 75th percentiles, respectively, and IQR (interquartile range) represent the difference between them.

b. Similar to **a** except showing conservation states and PhastCons score learned from the vertebrate CoV alignment.

c-d. Similar to **a-b**, respectively, except showing PhyloP score instead of PhastCons score.

e. Shown for each conservation state learned from the Sarbecovirus alignment (x-axis) is the fraction of bases overlapping PhastCons elements based on the same alignment (y-axis). Indicated by the

horizontal dashed line is the genome-wide coverage of the PhastCons element annotation. The exact coverage is reported below the line.

f. Similar to **e** except showing conservation states and PhastCons elements learned from the vertebrate CoV alignment.

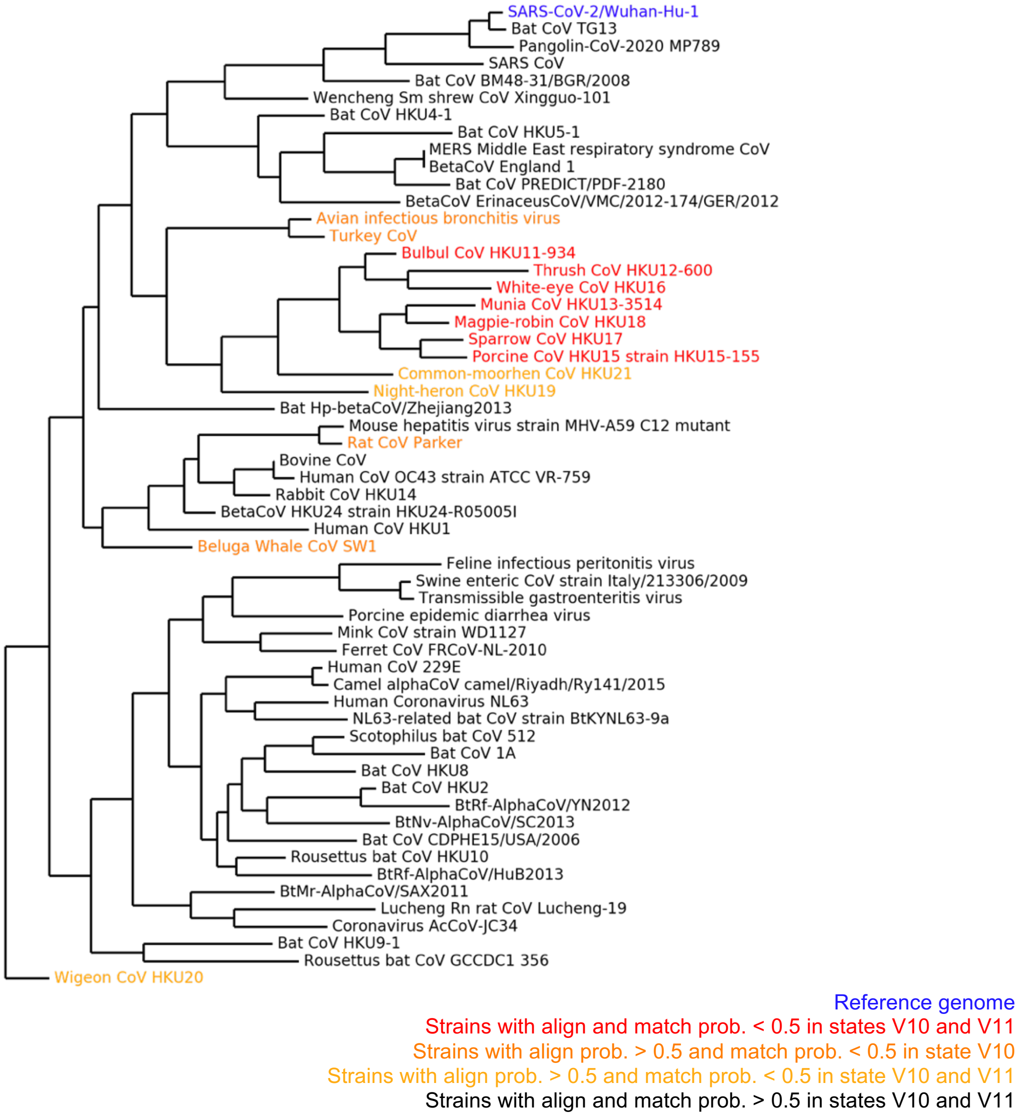

Supplementary Figure 5. Vertebrate CoV associated with states V10 and V11 in the phylogenetic tree of the vertebrate CoV alignment. Similar to **Fig. 3c** except strains colored according to their align and match probabilities in states V10 and V11. The strain colored in blue is the reference SARS-CoV-2 strain of the alignment, Wuhan-Hu-1. The strains colored in red are those with both align and match probabilities below 0.5 for both states V10 and V11, which include six CoV from avian hosts and a CoV from pig. The strains colored in orange are those with align probabilities above 0.5 and match probabilities below 0.5 for state V10. The strains colored in yellow are those with align probabilities above 0.5 and match probabilities below 0.5 for state V11. The remaining strains in black are those with align and match probabilities above 0.5 for both states.

a

Enrichment for SARS-CoV-2 mutations in states learned from the **Sarbecovirus** alignment

State	Enrichment for *nonsingleton* mutations			Enrichment for *all observed* mutations		
	Based on GW expectation	Corrected by nucleotide composition	Corrected by mutation type	Based on GW expectation	Corrected by nucleotide composition	Corrected by mutation type
S1	1.0	1.2	0.8	1.1	1.3	0.9
S2	1.2	1.4	0.9	1.1	1.3	0.8
S3	1.3	1.4	1.0	1.3	1.5	1.1
S4	1.4	**1.6**	1.1	**1.3**	**1.5**	1.1
S5	1.4	1.4	1.1	1.3	1.4	1.1
S6	**2.1**	**1.9**	**1.6**	**1.7**	**1.7**	**1.4**
S7	1.2	**1.3**	1.0	**1.2**	**1.3**	1.0
S8	1.5	**1.6**	1.2	**1.4**	**1.6**	1.2
S9	1.7	1.6	1.6	**1.8**	**1.7**	**1.7**
S10	1.5	1.5	1.3	1.2	1.2	1.1
S11	0.9	1.2	0.7	1.1	1.3	0.9
S12	**1.8**	**1.9**	1.4	1.3	1.4	1.0
S13	1.2	1.5	0.9	1.0	1.2	0.8
S14	1.0	1.2	0.8	1.1	1.2	0.9
S15	1.7	2.0	1.5	1.5	1.7	1.4
S16	1.3	1.3	1.1	1.1	1.2	1.0
S17	**0.7**	**0.6**	**0.8**	**0.8**	**0.7**	**0.9**
S18	**0.6**	**0.6**	**0.6**	**0.8**	**0.8**	**0.8**
S19	**1.3**	1.2	1.2	1.2	1.1	1.1
S20	1.0	1.3	0.8	1.0	1.2	0.9
S21	1.5	1.7	1.2	1.5	1.7	1.2
S22	0.9	1.0	0.7	1.1	1.2	0.9
S23	1.2	1.5	1.4	1.0	1.2	1.2
S24	1.5	1.6	1.5	1.4	1.5	1.5
S25	1.1	1.1	1.2	1.1	1.2	1.2
S26	**2.4**	**2.2**	**2.0**	**1.6**	**1.6**	**1.4**
S27	0.9	0.9	1.0	1.0	1.0	1.0
S28	**2.0**	**1.8**	**2.0**	**1.7**	**1.6**	**1.7**
S29	**2.4**	**2.2**	1.3	**2.1**	**1.9**	1.3
S30	0.0	0.0	0.0	0.0	0.0	**0.0**

b

Enrichment for SARS-CoV-2 mutations in states learned from the **vertebrate CoV** alignment

State	Enrichment for *nonsingleton* mutations			Enrichment for *all observed* mutations		
	Based on GW expectation	Corrected by nucleotide composition	Corrected by mutation type	Based on GW expectation	Corrected by nucleotide composition	Corrected by mutation type
V1	0.6	0.8	0.6	0.8	0.9	0.8
V2	1.2	1.1	1.0	1.1	1.1	1.0
V3	**1.7**	1.2	**1.4**	**1.4**	1.1	1.2
V4	1.0	1.2	0.9	1.0	1.2	0.9
V5	0.6	0.6	0.8	0.7	**0.6**	0.8
V6	1.0	0.9	0.9	1.0	0.9	1.0
V7	0.7	0.8	0.7	0.8	0.9	0.8
V8	1.4	1.2	1.3	1.2	1.1	1.1
V9	1.0	1.0	1.0	1.0	1.0	1.0
V10	**0.4**	**0.6**	**0.4**	**0.6**	**0.7**	**0.6**
V11	**0.2**	**0.2**	**0.3**	**0.3**	**0.3**	**0.4**
V12	1.0	1.1	1.1	1.0	1.0	1.0
V13	**1.5**	1.3	**1.5**	**1.4**	**1.3**	**1.4**
V14	1.2	1.1	1.2	1.2	1.1	1.2
V15	1.0	1.0	0.9	1.1	1.1	1.0
V16	1.1	1.1	1.1	1.1	1.2	1.1
V17	0.8	0.8	0.8	0.8	0.8	0.8
V18	1.0	0.9	1.0	1.0	0.9	1.0
V19	1.1	1.1	1.1	1.1	1.1	1.1
V20	**1.7**	**1.5**	**1.4**	**1.6**	**1.6**	**1.4**
V21	1.2	1.2	1.1	1.1	**1.1**	1.1
V22	1.1	1.1	1.1	0.9	1.0	1.0
V23	1.3	1.3	1.3	1.3	1.2	1.2
V24	1.1	1.1	1.2	0.9	0.9	1.0
V25	0.8	0.8	0.8	**0.8**	**0.8**	**0.8**
V26	0.7	0.7	0.9	**0.6**	**0.7**	0.8
V27	**0.2**	**0.2**	**0.3**	**0.4**	**0.4**	**0.5**
V28	1.3	1.3	1.3	**1.4**	**1.4**	**1.4**
V29	1.6	1.5	1.7	1.4	1.3	1.4
V30	**1.8**	**1.8**	**1.8**	**1.6**	**1.6**	**1.6**

Supplementary Figure 6. Conservation state enrichment for SARS-CoV-2 mutations.

a. Fold enrichment for SARS-CoV-2 mutations in conservation states learned from the Sarbecovirus model. Each row corresponds to a state. First column contains the state ID. State ID is shown in red if the state was significantly enriched for mutations in all six settings in which we computed enrichment, which are shown in the following six columns. State ID is shown in blue if the state was significantly depleted for mutations in all settings. Otherwise, state ID is shown in black. Second column contains fold enrichment values for nonsingleton mutations currently observed in SARS-CoV-2 mutations where the enrichment is computed as the ratio between the fraction of observed mutations among possible mutations in each state and the genome-wide (GW) fraction of observed mutations among possible mutations, as done in

Fig. 2b (**Methods**). Third column contains fold enrichment values for the same set of nonsingleton mutations except the enrichment is corrected by the nucleotide composition of the bases annotated by each state (**Methods**). Similarly, fourth column contains enrichment values for nonsingleton mutations corrected by the type (i.e. intergenic, synonymous, missense, nonsense) of the mutations annotated by each state (**Methods**). Fifth, sixth, and seventh columns are similar to second, third, and fourth columns except the enrichment values are computed based on all observed mutations instead of nonsingleton mutations. All mutations were reported by Nextstrain[1] based on sequences available on GISAID[2] (**Methods**). Each cell corresponding to an enrichment value is colored based on its value with blue as 0 (annotation not overlapping the state), white as 1 to denote no enrichment (fold enrichment of 1), and red as the maximum enrichment value in this table. A value is shown in bold if the associated two-sided binomial test p-value was significant at a 0.05 threshold after Bonferroni correction.

b. Similar to **a**, except based on states learned from the vertebrate CoV model. Row order in this figure do not have any correspondence to row order in **a**.

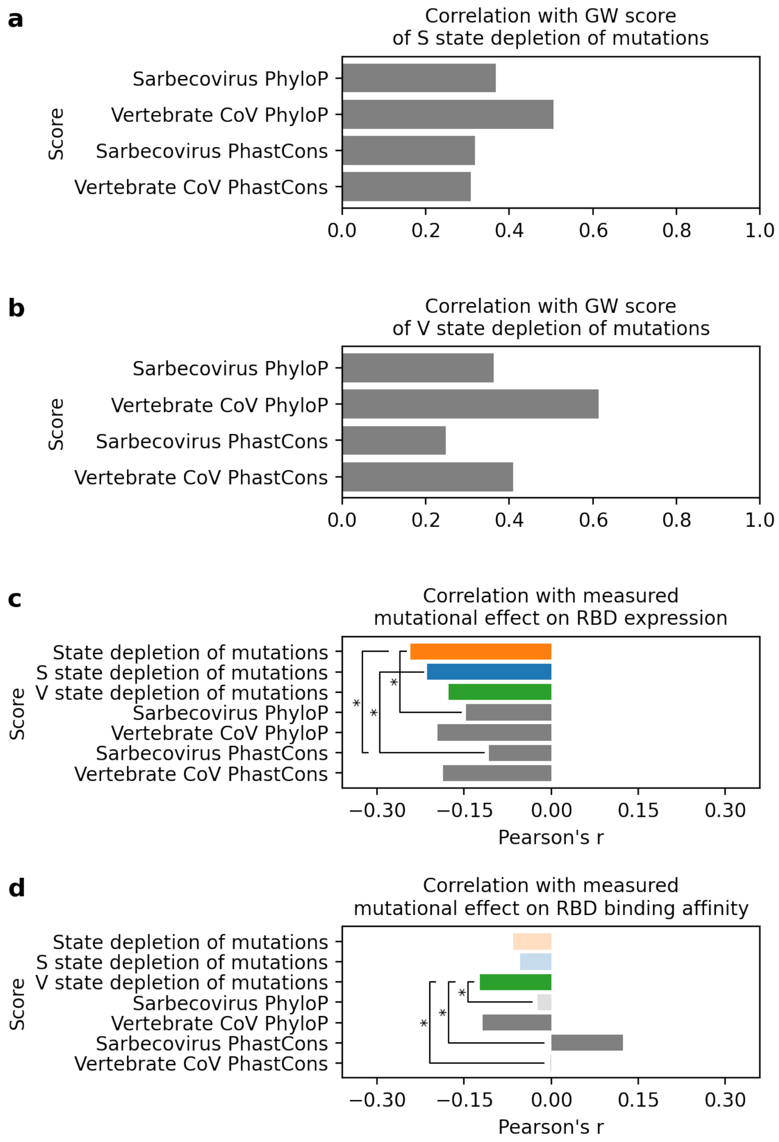

Supplementary Figure 7. Correlation with measured mutational effect for tracks based on state depletion of mutations and existing sequence constraint scores.

a. Bar graph showing correlation between our genome-wide (GW) score of depletion of mutations in conservation states from the Sarbecovirus model and four sequence constraint scores listed along the y-axis. The sequence constraint scores were based on either the Sarbecovirus or vertebrate CoV sequence alignment provided to ConsHMM using either PhastCons or PhyloP as the scoring method (**Methods**). A similar plot using the genome-wide score of depletion of mutations in states from both ConsHMM models instead of only the Sarbecovirus model is shown in **Fig. 4f**.

b. Similar to **a**, except using genome-wide (GW) score of depletion of mutations in conservation states from the vertebrate CoV model instead of the Sarbecovirus model.

c. Bar graph showing correlation between measured mutational effect on RBD expression and seven scores, which include three genome-wide scores based on conservation state depletion of mutations and four existing sequence constraint scores. Correlations computed with our scores based on both ConsHMM models, the Sarbecovirus model, and the vertebrate CoV model are shown in orange, blue, and green bars, respectively. Correlations computed with sequence constraint scores are shown in grey bars. Correlations with no statistical significance after Bonferroni correction by the total number of scores ($p < 0.05/7$) are shown in lighter colors (**Methods**). Black connecting lines and an asterisk are shown for pairs of a state-based score (colored bars) and an existing constraint score (grey bars) if at least one of the two scores has a statistically significant negative correlation and if the two scores also exhibit statistically significant difference in their correlations. Statistically significant difference in correlation was determined based on Zou's confidence interval test[6]. The test's confidence level was set to 0.996 (1 – 0.05/12) after Bonferroni correction by the number of pairs of a state-based score and a constraint score, where at least one score in the pair has a statistically significant negative correlation with mutational effect on RBD expression (**Methods**). Mutational effect on RBD expression was measured by the study referenced in **Fig. 4g** that conducted a deep mutational scanning of 3,819 nonsynonymous amino acid mutations in RBD[7]. To compute the correlations we restricted to the 1,215 mutations that were caused by single nucleotides and free of experimental measurements that were not determined (n.d.). A positive value indicates increased expression due to mutation and a negative value indicates decreased expression. A subset of the correlations shown here are also shown in **Fig. 4g**.

d. Similar to **c**, except showing measured mutational effect on RBD binding affinity instead of expression and using confidence level of 0.992 (1 – 0.05/6) for Zou's confidence interval test given six pairs of correlations to compare (**Methods**).

References

1. Hadfield, J. *et al.* Nextstrain: real-time tracking of pathogen evolution. *Bioinformatics* **34**, 4121–4123 (2018).
2. Elbe, S. & Buckland-Merrett, G. Data, disease and diplomacy: GISAID's innovative contribution to global health. *Glob. Challenges* **1**, 33–46 (2017).
3. van Dorp, L. *et al.* Emergence of genomic diversity and recurrent mutations in SARS-CoV-2. *Infect. Genet. Evol.* **83**, 104351 (2020).
4. Gussow, A. B. *et al.* Genomic determinants of pathogenicity in SARS-CoV-2 and other human coronaviruses. *Proc. Natl. Acad. Sci.* **117**, 15193–15199 (2020).
5. The UniProt Consortium. UniProt: a worldwide hub of protein knowledge. *Nucleic Acids Res.* **47**, D506–D515 (2019).
6. Zou, G. Y. Toward using confidence intervals to compare correlations. *Psychol. Methods* **12**, 399–413 (2007).
7. Starr, T. N. *et al.* Deep mutational scanning of SARS-CoV-2 receptor binding domain reveals constraints on folding and ACE2 binding. *Cell* **182**, 1295-1310.e20 (2020).
