# Supplementary material for: Single-nucleotide conservation state annotation of the SARS-CoV-2 genome
Source: Commun Biol. 2021 Jun 3;4:698. doi: 10.1038/s42003-021-02231-w (PMC8175581; doi:10.1038/s42003-021-02231-w)
Supplement: Supplementary file 3 — Description of Additional Supplementary Files [file 42003_2021_2231_MOESM3_ESM.pdf]

Description of Additional Supplementary Files

File name: Supplementary Data 1

Description: SARS-CoV-2 genome-wide tracks of ConsHMM conservation state annotation and separately mutation depletion score based on significant enrichment and depletion of nonsingleton SARS-CoV-2 mutations.

a. wuhCor1.mutDepletionConsHMM.bed:

BedGraph format file with its four columns corresponding to chromosome, start, end, and mutation depletion score based on significant enrichment/depletion of nonsingleton SARS-CoV-2 mutations based on all conservation states.

b. wuhCor1.mutDepletionSarbecovirusConsHMM.bed:

Similar to **a** except the last column reports mutation depletion score based on significant enrichment/depletion of nonsingleton SARS-CoV-2 mutations based on states learned from the Sarbecovirus alignment.

c. wuhCor1.mutDepletionVertebrateCoVConsHMM.bed:

Similar to **a**, except the last column reports mutation depletion score based on significant enrichment/depletion of nonsingleton SARS-CoV-2 mutations based on states learned from the vertebrate CoV alignment.

d. wuhCor1.sarbecovirusConsHMM.segments.bed:

BED format file with its four columns corresponding to chromosome, start, end, and state assignment based on the Sarbecovirus ConsHMM model.

e. wuhCor1.vertebrateCoVConsHMM.segments.bed:

Similar to **d**, except the last column reports state assignment based on the vertebrate CoV ConsHMM model.

f. wuhCor1.sarbecovirusConsHMM.bed:

Similar to **d**, except in BED format that is more suitable for viewing on a genome browser.

g. wuhCor1.vertebrateCoVConsHMM.bed:

Similar to **e**, except in BED format that is more suitable for viewing on a genome browser.

File name: Supplementary Data 2

Description: Source data for Fig. 2a-b, 3a-b, 4a-d,f-g, and Supplementary Fig. 7.
